# A Complex Investigation of LATP Ceramic Stability and LATP+PVDF Composite Membrane Performance: The Effect of Solvent in Tape-Casting Fabrication

**DOI:** 10.3390/membranes13020155

**Published:** 2023-01-26

**Authors:** Zainab Waris, Nikita O. Akhmetov, Mariam A. Pogosova, Svetlana A. Lipovskikh, Sergey V. Ryazantsev, Keith J. Stevenson

**Affiliations:** 1 Center for Energy Science and Technology, Skolkovo Institute of Science and Technology, 121205 Moscow, Russia; 2 Department of Chemistry, Lomonosov Moscow State University, 119991 Moscow, Russia

**Keywords:** redox flow batteries, composite membranes, ionic conductivity, structural stability

## Abstract

Redox flow batteries (RFBs) are a prospective energy storage platform to mitigate the discrepancy between barely adjustable energy production and fluctuating demand. The energy density and affordability of RFBs can be improved significantly through the transition from aqueous systems to non-aqueous (NAq) due to their wider electrochemical stability window and better solubility of active species. However, the NAqRFBs suffer from a lack of effective membranes with high ionic conductivity (IC), selectivity (low permeability), and stability. Here, we for the first time thoroughly analyse the impact of tape-casting solvents (dimethylformamide—DMF; dimethylsulfoxide—DMSO; N-methyl-2-pyrrolidone—NMP) on the properties of the composite Li-conductive membrane (Li_1.3_Al_0.3_Ti_1.7_(PO_4_)_3_ filler within poly(vinylidene fluoride) binder—LATP+PVDF). We show that the prolonged exposure of LATP to the studied solvents causes slight morphological, elemental, and intrastructural changes, dropping ceramic’s IC from 3.1 to 1.6–1.9 ∙ 10^−4^ S cm^−1^. Depending on the solvent, the final composite membranes exhibit IC of 1.1–1.7 ∙ 10^−4^ S cm^−1^ (comparable with solvent-treated ceramics) along with correlating permeability coefficients of 2.7–3.1 ∙ 10^−7^ cm^2^ min^−1^. We expect this study to complement the understanding of how the processes underlying the membrane fabrication impact its functional features and to stimulate further in-depth research of NAqRFB membranes.

## 1. Introduction

**Figure 1:**
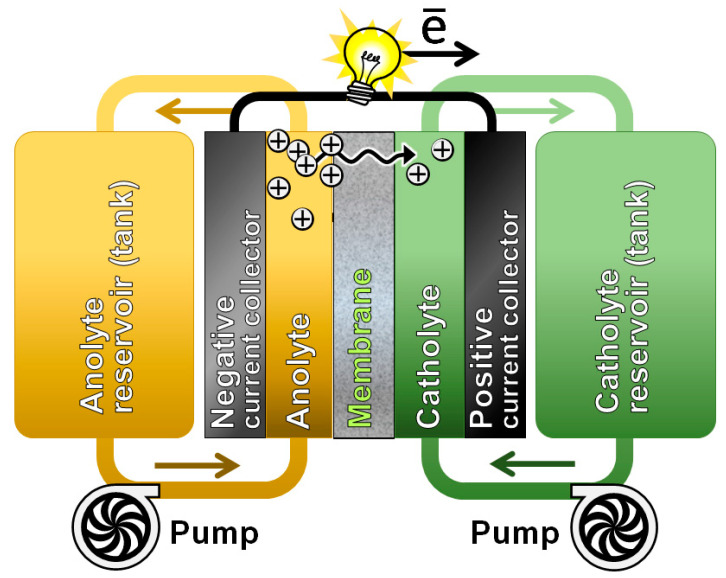
Schematic of a typical redox flow battery.

A drastic increase in the world’s population, the improvement of people’s lifestyles, and the rapid development of digital technologies have been continuously enlarging the total energy demand. Unfortunately, contemporary energy systems, both conventional and alternative, suffer from the discrepancy between the levels of energy generation and consumption. This leads to an extraordinary amount of energy overproduction, and it impedes the broad application of renewable technologies. Anticipating a possible global energy crisis, this situation urgently necessitates the need to develop and implement energy storage capacities into the existing electricity supply system [1]. Acting as buffers, these capacity units would follow the fluctuating cycles of actual energy demand by lowering the level of conventional energy generation and adapting the variable output from alternative energy sources. Among other energy storage solutions, redox flow batteries (RFBs) are promising candidates to be applied for this purpose (Figure 1) [2,3,4,5]. The ability to be rapidly recharged by changing electrolyte tanks, independent control of power and energy, and durability are only some of the RFB’s benefits in the backdrop of traditional Li-ion batteries used for stationary applications [2]. Nowadays, aqueous vanadium-based RFBs are the most developed class that has already been used for large-scale energy storage purposes [6,7,8]. However, they still suffer from the low active species solubility and operational voltage (1.4 V) limited by water decomposition that has resulted in low energy density (30–50 vs. 300 Wh L^−1^ for conventional Li-ion technology). Alternatively, the use of non-aqueous (NAq) electrolytes can broaden the operating voltage window and enhance solubility of active moieties, which would significantly boost RFB’s power and catalyze their implementation as energy storage devices.

One of the key components of RFBs is an ion-conductive solid membrane (sometimes referred to as a separator) [9,10,11,12] that maintains the charge balance in the cell [13,14,15]. Impacting RFB’s cycling stability and total cell resistance, the membrane strongly contributes to battery’s power density, durability, and efficiency. This moves membrane-related topics to the top of RFB’s investigation interests. In fact, it is still challenging to come up with a scalable membrane concept that would meet the full set of requirements. For instance, the membrane’s ionic conductivity (IC) in NAq electrolyte is still not high enough to approach the current densities of vanadium RFBs [16]. Besides, the membrane for NAqRFBs should provide low active species crossover, high thermal and chemical stability, and affordability [17,18]. Overall, the problem of membranes for NAqRFBs still remains unsolved, so more efforts should be devoted to a material design, physicochemical and electrochemical investigation, and life testing of membranes within the RFB prototypes.

Specific “filler inside matrix” functional composites were recently tested as ion-conductive membranes for both flow and solid-state batteries [19,20,21,22,23]. The components for such composite membranes were chosen the way in which they combine high IC from one side (filler) as well as stability, flexibility, and non-permeability from another side (matrix). Among the plenty of different compounds, such as garnets [24], NASICON-type ceramics [25], and perovskites [26] as fillers, and poly(vinylidene fluoride) (PVDF) [27], polyethylene oxide [28], and polyacrylonitrile [29] as matrices, we considered the NASICON ceramics together with PVDF to be the most promising combination. When the former is well-conductive and relatively stable, the latter is inert, flexible, and easily processed [30].

Since the beginning of our research activities, the path toward the desired lithium-conductive composite membrane has not been easy as we intended to develop the full-cycle synthesis routine. First, we designed the solid-state synthesis of Li_1.3_Al_0.3_Ti_1.7_(PO_4_)_3_ (LATP) ceramic of the pure NASICON-type phase with high IC (above 4 ∙ 10^−4^ S cm^−1^) but severe sensitivity to moisture that had never been characterized before [31,32,33]. From now on, we additionally expected the introduction of the inert matrix to protect the LATP filler from the ambient humidity and preserve its originally high IC. Then, we reported our first version of the tape-casting fabrication route of composite LATP+PVDF membranes [34] along with their general electrochemical properties and primary performance in organic flow and Li-hybrid cells [35,36]. We showed that the fabricated membranes demonstrated ultimate stability to metallic Li, high IC (3.4 ∙ 10^−4^ S cm^−1^), good integrity, but relatively high permeability to (2,2,6,6-tetramethylpiperidin-1-yl)oxyl (TEMPO)—a model redox active compound [35]. Based on the results collected, we reasonably expected the membrane’s porosity to be the main reason behind its permeability and cell’s capacity decay. To resolve this issue, relying on available publications, we consider the following factors to be the most impactful in our case: a tape-casting solvent, fabrication conditions, especially temperature and humidity, and a ceramic-polymer interface [37,38,39].

In this study, we work with the popular casting solvents suitable for a proper dissolution of PVDF, namely, dimethylformamide (DMF), dimethylsulfoxide (DMSO), and N-methyl-2-pyrrolidone (NMP), and for the first time thoroughly investigate their impact on the LATP+PVDF membrane and, specifically, the LATP ceramic filler within. The entire work was categorized into two parts, where a solid instrumental basis was applied to study the materials, including: X-ray diffraction (XRD), attenuated total reflectance Fourier-transform infrared spectroscopy (ATR-FTIR), scanning electron microscopy (SEM), energy dispersive X-ray spectroscopy (EDX), electrochemical impedance spectroscopy (EIS), and cyclic voltammetry (CV). Previously, we showed the instability of LATP toward water led to drastic IC losses, along with structural and morphological changes, mostly due to the elution of lithium ions [33]. The NAq solvents applied in this study possess twice lower polarity compared to water; the dielectric constants are: water 80.1, DMSO 46.7, DMF 36.7, and NMP 32.2 [40]. Due to this reason, we do not expect such an ionic and insoluble compound as LATP to possess the same level of sensitivity to the dipolar aprotic solvents (DAs) as for the water case [41]. Nevertheless, some dissociation of complex phosphate cannot be fully excluded as the solvents have a good ability to solvate lithium ions [41]. Taking into account this and the crucial role of lithium loss [33], we expect that DMF, DMSO, and NMP can actually affect LATP in a similar way to water but to a lower extent.

Here we need to take into account two important aspects: first, the tape-casting fabrication approach implicates a prolonged immersion of membrane components to the casting solvent; second, the LATP filler contributes the most to the IC of the final composite. Therefore, we should investigate if there is any negative impact of NAq solvents on LATP properties. For this reason, in the first part of our current research, we analyze the LATP ceramic’s crystal structure, morphology, and IC, comparing the values collected before and after static soaking of dense ceramics in the investigated solvents. In the second part, we continue the structural evaluation of LATP as a part of fabricated composites and study the final membranes’ parameters: morphology, IC, and permeability towards TEMPO species. We hope the results obtained in this article will shed the light on how the tape-casting fabrication process and, specifically, a solvent impact the properties of both ceramic filler and composite membrane. We believe that the precise analysis of polymer-ceramic class of membranes reported here will bring it closer to a wide implementation in RFBs and boost up the global transition to an environmentally benign energy generation.

## 2. Materials and Methods

### 2.1. Synthesis of Ceramic

The two-step solid-state method previously described in our recent publication [33] was used to synthesize the NASICON-based LATP ceramics. Briefly, NH_4_H_2_PO_4_ (98%, Alfa-Aesar, Tykyo, Japan), TiO_2_ (99%, Sigma-Aldrich, Taufkirchen, Germany), and Al_2_O_3_ (decomposed from Al(NO_3_)_3_ ∙ 9H_2_O, >97%, RusChem, Saint-Petersburg, Russia) were weighted in a stoichiometric ratio. Li_2_CO_3_ (99%, Sigma-Aldrich, Santiago, Chile) was taken with the 5% excess to compensate the loss of lithium during the high temperature annealing. In the first step, all the reagents were manually mixed and milled in an agate mortar. After that, several milliliters of isopropanol were added, and the milling was continued until its complete evaporation. The obtained powder was then placed into the alumina crucible with burnable separator installed preliminarily and heated to 750 °C for 14 h (holding for 3 h) in a muffle furnace (Nabertherm, Lilienthal, Germany) followed by air quenching and natural cooling inside the crucible. In the second step, the sample was ground the same way as in the first step but with addition of 5 wt% polyethylene glycol (M_w_ ~1500, Sigma-Aldrich, St. Louis, MO, USA) in the form of isopropanol solution during continuous milling until dry. After, the powder was separated into the portions of 0.25 g each and pressed (5000 psi) to pellets (diameter of 9.85–10.00 mm and thickness of 1.54–1.58 mm). Finally, the pellets were sintered at 850 °C for 14 h (holding for 1.5 h) and quenched rapidly by throwing on the surface of aluminum foil. The ceramic pellets were either immediately analyzed or placed inside the Ar-filled glove box (c(H_2_O) < 10 ppm, c(O_2_) < 10 ppm; MBraun, Garching bei München, Germany), where they were sealed in vacuum pouches and stored for later use. The complete ceramic synthesis scheme can be seen in Figure S1, Supplementary Materials.

### 2.2. Fabrication of Composite Membranes

The composite membranes were fabricated using a simple tape-casting method (Figure S2, Supplementary Materials) close to the previously reported [35] but with several minor changes (casting temperature, heating bath, drying atmosphere etc.). The component ratios of LATP:PVDF and PVDF:solvent were optimized in our previous publications to achieve the suitable IC/permeability trade-off and for the ease of the casting procedure; the ratios equal 45:55 and 15:85, respectively [34,35]. The as-synthesized ceramic pellets were manually ground in the agate mortar, followed by the intense ball-milling (SPEX 8000, Metuchen, NJ, USA) for 90 min in an agate vial with one milling sphere to achieve the mean particle size of about 1 μm, according to our previous report [35]. Simultaneously, 0.5 g of PVDF (M_w_ ~534000, Sigma-Aldrich, St. Quentin Fallavier Cedex, France) was dissolved under magnetic stirring (400 rpm) in 3 mL of either DMF (≥99.8%, RusChem, Russia), DMSO (≥99.7%, Acros Organics, Branchburg, NJ, USA), or NMP (≥99.5%, Acros Organics, NJ, USA), all preliminarily dried with 3 Å sieves until c(H_2_O) < 25 ppm. Then, a calculated amount of ceramic powder was added into the solution. The mixing was performed at 60 °C (putting a vial with the solution inside an Al-foil bath) and 1400 rpm for 4 h, followed by the degassing step (standing still at 23 °C for 12 h). After that, the membranes were cast using the film applicator (Zehntner ZAA 2300, Sissach, Switzerland) on the preheated to 70 °C clean glass substrate (the blade gap was 400 µm, gliding rate was 15 mm s^−1^). In the final step, the as-cast samples were dried for 1 h at 80 °C and atmospheric pressure.

### 2.3. Soaking Experiments

**Figure 2:**
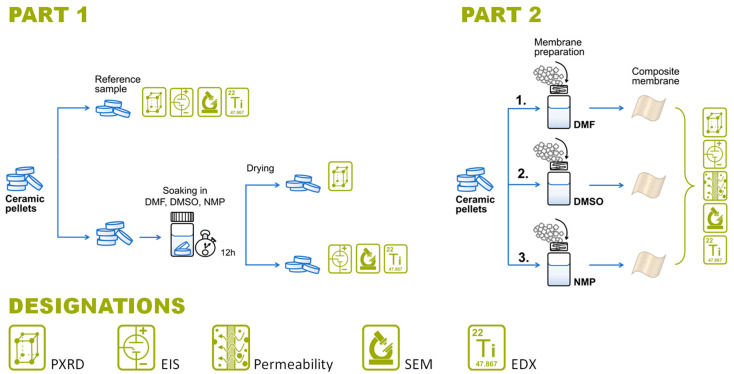
General roadmap of LATP ceramic and LATP+PVDF membrane investigation.

To analyze the impact of solvents applicable for the membrane casting (DMF, DMSO, and NMP) on LATP behavior, we statically soaked the ceramic pellets in each of them and subsequently compared the characteristics of initial and treated LATPs (Figure 2). The soaking was performed in the following way: 5 LATP pellets of 0.25 g each (4 samples for EIS; 1 for XRD and SEM) were placed in 15-mL glass vials contained 3 mL of the certain solvent for 12 h. After the soaking, the samples were taken out and dried for 1 h at room temperature under dynamic vacuum. The dried LATP samples, as well as untreated ones (reference samples), were examined via the thorough instrumental analysis, including phase and chemical composition studies (XRD), morphology investigation (SEM), and IC measurements (EIS).

### 2.4. Structure, Composition, and Morphology Analysis

XRD was performed for the investigation of the ceramic’s structure using the Bruker D8 Advance (Billerica, MA, USA) diffractometer equipped with the CuKα_1,2_ radiation set-up, and a LynXeye XE detector (1 mm fixed divergence slit, the soller slits of 2.5°, 30 rpm sample holder rotation, 2θ range from 10° to 120°, θ step of 0.01°, ambient conditions, room temperature). The data were collected at the final patterns' maximum intensities for about 100,000 counts. The Rietveld method was applied to analyze the crystal structures using the JANA 2006 software package [42]. Similar to our previous articles [31,33], we consider the LATP structural changes through the glance of selected intrastructural units—polyhedrons surrounding positions of lithium, phosphorus, and the shared position of two main cations aluminum and titanium. Particularly, the changes in polyhedrons’ volume are of main interest, as they indicate the chemical changes [31,33]. For XRD analysis, the LATP pellets were milled manually in the agate mortar to produce a fine powder. The powder was spread on the flat side of the sample holder with the help of isopropanol to make a thin and uniform layer. For composite membrane analysis, samples were fixed on the flat side of the sample holder by a roentgen-amorphous petroleum jelly.

ATR-FTIR spectra of pristine PVDF powder and the LATP+PVDF membranes were measured using Bruker Alpha II (MA, USA) spectrometer equipped with a diamond ATR crystal and a KBr beamsplitter. Data were collected in the 4000–400 cm^−1^ range within the 4 cm^−1^ resolution and signal averaging by 20 scans.

The Thermo Fisher Scientific FEI Quattro S (Waltham, MA, USA) microscope was utilized for SEM and EDX imaging membranes’ morphology and mapping elemental distribution; 2 kV acceleration voltage, 0.11 nA beam current, and a low-vacuum detector provided a highly resolved morphology of the top and cross-section views of membranes. The cross-section imaging was performed using brittle crack in liquid nitrogen [43].

### 2.5. Electrochemical Analysis

The Metrohm Autolab potentiostat-galvanostat PGSTAT302N (Metrohm AG, Barendrecht, Netherlands) was used to perform EIS. The measurements were taken at frequencies ranging from 1 MHz to 0.1 Hz and an amplitude of 0.1 V with the application of a custom-made electrochemical cell [32]. Spectra for each sample were recorded five times for better statistics and error evaluation. The mean square root approximations were performed within the Metrohm Autolab NOVA software. A Kramers–Kronig relationship (χ^2^ ~10^−5^–10^−7^) was used to determine the quality of the obtained data. For LATP analysis, the surface of pellets was covered with 30 nm thick gold layers on both sides through the magnetron sputtering (Q150T S/E/ES Sample Preparation System; Quorum, Lewes, UK), which served as blocking electrodes. For the measurements of composite membranes, the samples were soaked in the solution of 1.0 M LiClO_4_ (99.99%, Sigma Aldrich, USA) in propylene carbonate (PC, ≥99.7%, Sigma-Aldrich, Budapest, Hungary) for 18 h prior to impedance tests. Then, coin cells with stainless steel blocking electrodes and the soaked membrane placed in-between were assembled and tested.

Membranes’ permeability coefficients were identified by CV using the PalmSens4 (GA Houten, Netherlands) galvanostat/potentiostat (in accordance with the methodology described in our previous study [35]). Briefly, the custom-made diffusion cell (Figure S3, Supplementary Materials) contains two compartments: the right one was filled with 0.5 M TEMPO (98%, Sigma-Aldrich, Germany) dissolved in the supporting electrolyte (SE, 1 M LiClO_4_ in PC), while the left one was equipped with a three-electrode system and contained SE only. In such a set-up, we observe the peak current growth on CV scans with time attributed to diffusion of TEMPO molecules from the right compartment to the left through a membrane placed in-between. The three-electrode system applied consisted of: the working electrode made of a Pt wire (1.6 mm diameter); the counter electrode made of 3-mm glassy carbon; the reference electrode made of a 0.5-mm Ag wire. CV recording was performed at 23 °C, potential range of −0.2–0.8 V vs. Fc/Fc^+^, and 0.05 V s^−1^ scan rate. A mathematical justification of the used electrochemical methods can be seen in Section S2, Supplementary Materials.

## 3. Results

### 3.1. Investigation of Ceramic’s Stability toward Casting Solvents

To be industrially prospective, a membrane should not only possess promising initial performance (a combination of optimal structural, morphological, and electrochemical properties) but maintain it throughout the whole membrane’s life cycle. In our case, the membrane is composed of PVDF polymer matrix and LATP inorganic filler, where the latter contributes the most to the final composite’s IC [35]. Therefore, along with the functional behavior of LATP+PVDF, the stability of LATP itself is of great interest to our study. Previously, we briefly described the structural changes as well as sufficient elemental and IC losses of the LATP ceramic in contact with water [33], despite the widely spread impression of its water sustainability [44]. Therefore, one cannot exclude the degradation scenario during the prolonged contact of LATP with non-aqueous solvents, which eventually happens throughout the tape-casting membrane fabrication lasting for about 24 h in total. So, in this section, we carefully analyze the LATP stability toward the most popular and affordable PVDF casting solvents, namely, DMF, DMSO, and NMP. This will show the way each of them influences LATP, which, in turn, would define several properties of the whole composite membrane.

In order to estimate the functional, structural, and morphological stability of LATP toward the studied solvents, we performed its soaking treatment. This treatment was applied to the dense ceramic pellets, allowing us to compare them with a reference sample, so that its properties would serve as starting points. Here we need to underline that we acknowledge the differences between the ceramic-solvent interfaces in ceramic soaking and membrane preparation cases where the ceramic used is a milled powder. We admit that the impact of the solvents on LATP features observed under the conditions used in this chapter might be weaker than in the case of a full membrane fabrication routine. Yet, immersion of the dense ceramics is almost the only way to properly compare data on the LATP-solvent stability. Otherwise, if applied in a powdered form, LATP should be collected after immersion, pressed, and annealed to obtain a dense ceramic suitable for ionic conductivity measurements. Moreover, this multistep procedure would introduce many unmeasurable factors and deprive us of the proper reference sample. Nevertheless, we maintained other non-destructive conditions of the soaking experiment equivalent to the membrane fabrication: processing at room temperature; soaking duration as in the degassing procedure (12 h).

#### 3.1.1. LATP’s Microstructure

Following a general overview of the pristine LATP ceramics (Figure S4a, Supplementary Materials), one can observe a dense polycrystalline microstructure: tightly placed well-formed grains (average size of 1–5 µm) with defined edges and no evident impurities. The EDX mapping provided in Figure S5, Supplementary Materials, demonstrates the uniform elemental distribution, so we consider the initial LATP ceramic well-crystallized and pure. After soaking in the DMF, DMSO, and NMP solvents, LATP, at first sight, keeps its initial microstructural features (Figure S4b,d, Supplementary Materials): one can still see well-defined and distinguishable grains. It is possible to see little round-like particles disseminated around cavities and grain edges, especially in the DMF and NMP cases. Referring to the elemental mapping (Figure S5, Supplementary Materials), the unevenness of elemental distribution becomes detectable for phosphorus ion, which now leans toward intergranular spaces, especially evident for the DMF case. Besides, the elemental analysis showed a significant Al loss in the edges of ceramic grains after soaking in DMF—a molar fraction of Al decreased from 0.2 to 0.1 (Table S1, Supplementary Materials). These facts hint at a specific ceramic-DMF interaction, which may cause LATP structural deviations at the grain edges. The elemental distribution of ceramic soaked in DMSO and NMP showed a 10–20% deviation of Al-Ti ratio from the pristine sample (Table S1, Supplementary Materials), which is acceptable in LATP elemental analysis [33].

**Figure 3:**
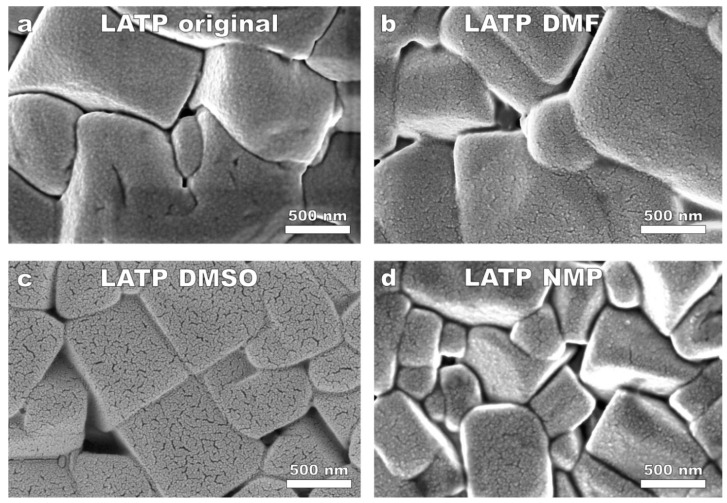
SEM images of (**a**) pristine LATP ceramic and that after soaking in (**b**) DMF, (**c**) DMSO, and (**d**) NMP.

However, taking a closer look at the magnified SEM images of the same LATP samples (Figure 3), we can truly observe the changes occurred in grains. As for the pristine LATP ceramic (Figure 3a), we see grains with barely visible roughness on their sides, quite sharp edges, and tight and well-visible intergranular contacts. When the solvents were applied, a grain surfaces’ roughness dramatically increased (Figure 3b,d): the grains became eroded and covered with a net of nanocavities, especially, for DMSO. Interestingly, the comparative estimation of the erosion severity correlates with the polarity level of the solvents: DMSO exhibits the highest dielectric constant of 46.7, while DMF and MNP possess comparable values of 36.7 and 32.2, respectively. This might cause DMSO to promote slightly stronger partial dissociation of LATP represented in the etched surface of ceramic grains. Thus, the intergranular spaces are no longer the same: instead of fine and contrast contacts between grains, we see wider cavities either still contrast (Figure 3d—NMP case, probable slight dissolution of material) or much less contrasted (Figure 3b,c—DMF and DMSO, intragranular cavities likely contain a product of ceramic-solvent interface). Some of these morphological changes are comparable with those described in our recent publication devoted to the degradation of LATP in water [33]. After being similarly immersed in deionized water, the LATP ceramic also showed the more rounded grain edges, wider and less contrast intergranular cavities, and formation of nanoparticles. The major difference though between the impacts of dipolar aprotic solvents (DAs) and water cases is the grains’ surface erosion that was not detected in the water study [33].

Based on the data above, we can reasonably conclude that the LATP ceramic does undergo some visible morphological changes during the prolonged contact with the DA solvents applied. This, in turn, alluded to the existence of specific ceramic-solvent interaction (and corresponding interface formation) in our systems. For a deeper understanding of the solvents’ effect, we further analyzed LATP’s crystal structure and explored the changes of its main functional feature—IC.

#### 3.1.2. Crystal Structure and IC

In order to analyze the behavior of LATP’s crystal structure, we followed the approach described in our study on the LATP stability toward air and argon [31]. In that work, we did not only consider the unit cell dimensions, but also discussed the intrastructural units—the polyhedra that form the surroundings of LATP’s cations. This approach enables an indirect analysis of the cationic changes barely detectable directly (e.g., related to Li^+^ ions). For instance, based on the changes in volumes of specific polyhedrons around lithium positions, we concluded the lithium instability at the Li(1) position to be the main driving force for the whole LATP degradation process in both structural and IC aspects [31]. Here, we will operate with polyhedra volumes defined and calculated in exactly the same manner. All characteristic values were considered in comparison with the reference samples (i.e., untreated, pristine LATP) synthesized within the same synthetic series. Particular data collected for all samples under investigation are provided in Section S4, Supplementary Materials.

As one can see (Figure S6, Supplementary Materials), the prolonged immersion of LATP ceramics into all the studied solvents led to the visible shrinkage of unit cell volume (*V*) mainly affected by the *c* dimension decrease. Similar behavior had been described for the case of LATP degradation in air and argon atmospheres [31]. Even though one may consider the changes of *V* as negligibly small (0.16% for DMF and 0.08% for both DMSO and NMP), they are comparable with the values observed for air degradation (0.12%), which was accompanied with the IC losses of about 75%. Hence, even this first observation can indicate the presence of structural changes associated with the IC fall.

Further, we monitored the changes in the structural unit that most likely correlates with LATP’s IC—the [Li(1)O_6_M_2_] polyhedra. In Figure S6, Supplementary Materials, one can observe small and controversial changes in its polyhedron volume: it gains 1.02% and 0.88% for DMF and NMP solvents, respectively, while losing 0.19% in the case of DMSO. It is worth mentioning that the polyhedron’s volume changes are quite close to those observed in the study of air impact (within 0.70%), where they had a good correlation with the IC trend during the long-term LATP storing experiment [31]. So, the interaction between LATP ceramics and applied aprotic solvents impacts the IC-sensitive intrastructural center of [Li(1)O_6_M_2_] to an extent comparable with the 100-day air storage.

**Figure 4:**
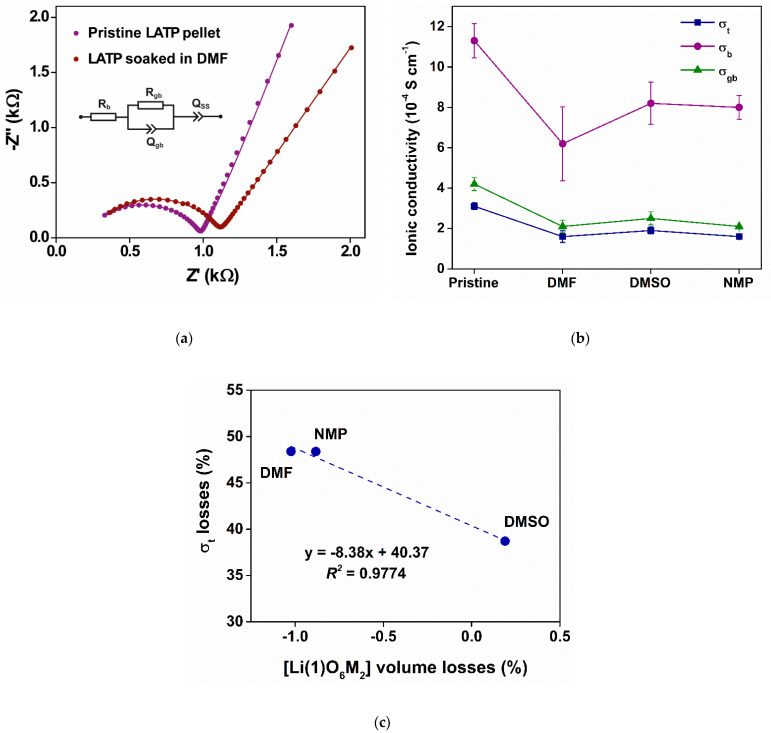
(**a**) Example Nyquist plots of as-synthesized LATP ceramic and LATP after soaking in DMF, dots relate to experimental data and lines−to the fitting curves, inset−for which an equivalent circuit used for the approximation; (**b**) total (*σ_t_*), bulk (*σ_b_*), and grain boundary (*σ_gb_*) IC values of pristine LATP and that after soaking in DMF, DMSO, or NMP; (**c**) correlation between volume of [Li(1)O_6_M_2_] intrastructural polyhedron and *σ_t_* of LATP ceramic.

The example of experimental Nyquist plots, fitting curves, applied equivalent circuit, and processing details can be found in Figure 4a as well as in Section S2, Figure S9, and Table S3, Supplementary Materials. In brief, the total ionic resistance (*R_t_*) of the LATP ceramic is composed of the bulk (*R_b_*, semicircle intercept with Z’) and grain boundary (*R_gb_*, the semicircle diameter) resistances [32]. The calculated bulk (*σ_b_*), grain boundaries (*σ_gb_*), and total (*σ_t_*) ICs for pristine and soaked ceramic are presented in Figure 4b. From our analysis, the *σ_t_* losses of the soaked samples approach a half of initial values: 48.4% for DMF and NMP and 38.7% for DMSO. These values are lower than that from the water experiment (64% *σ_t_* losses) and almost correspond to the 1-month storage in ambient atmosphere. The correlation between the changes of IC and [Li(1)O_6_M_2_] polyhedron’s volume illustrated in Figure 4c shows that the alterations at the sensitive Li(1) position evidently have a direct impact on total IC—yet can be considered as one factor among many others. Thereby, as in the previous cases (air/argon storage and water soaking), solvents investigated in this work have a strong impact on lithium in the initial structure.

The DMF, DMSO, and NMP solvents are totally organic and were preliminarily dried to eliminate the impact of water—an aggressive media for LATP [33]. Due to this reason, we expected a low-to-no impact on LATP’s structure and IC, but even for the delicate treatment (static soaking experiment), we clearly see the morphological, structural, and IC degradation. Unfortunately, there is a lack of information related to the stability of inorganic water-insoluble phosphates toward DMF, DMSO, and NMP in particular, and toward DA solvents in general, which necessitates a separate and thorough study. But at this point, we can only consider very limited reports that can shed the light on the processes behind the changes observed.

In DA solvents, ionic species are usually solvated and can dissociate, but to a smaller extent than in water. Anions are less solvated by DAs (compared to cations) due to their aprotic nature and nucleophilicity. Li^+^ ion has good solubility in DA (yet lower than in water) and can promote the dissociation of the whole compound. Moreover, the larger anion the salt has, the better solvation it might receive. There are rare mentions of K_3_PO_4_ dissolution in DMSO and DMF as well as anhydrous H_3_PO_4_ in DMF [45], so even phosphate anions might get a considerable solvation. In addition to this, DAs are known to be efficient ligands for such ions as Ti^4+^ and Al^3+^ [41]. Overall, based on the collected experimental and literature data, we can reasonably expect LATP to get partially solvated by DMF, DMSO, and NMP, which, in turn, suppresses the main ceramic’s functional feature —IC.

Overall, LATP does degrade in contact with the studied solvents, although the total IC of LATP was still at acceptable level (>10^−4^ S cm^−1^). The influence of all the studied solvents on LATP was not too severe to unambiguously highlight or exclude anyone from the list. It encouraged us to continue investigating ceramic’s properties at the next level: after blending LATP within a polymer (PVDF) matrix and forming a composite membrane.

### 3.2. Structural, Morphological, and Electrochemical Properties of LATP+PVDF Composite Membranes

#### 3.2.1. LATP’s Crystal Structure within the Composite Membrane

Stand-alone ceramic ionic conductors would unlikely be widely used as battery electrolytes due to their fragility, permeability, and instability to metallic lithium. Blending fine ceramic particles within a polymer matrix is one of the approaches to obtain a solid electrolyte with the compromising properties—high IC (impact of ceramic filler) and enhanced flexibility and stability (impact of polymer matrix). Continuing thorough investigation of LATP stability, here we focus on composite membranes, LATP+PVDF, fabricated using the DMF, DMSO, and NMP solvents.

Although we have just concluded on the solvent influence on LATP, the polymer surrounding it and the fabrication process in general can introduce additional changes to the ceramic. Moreover, the crucial property of membranes for RFBs is their permeability to species involved in electrochemical reactions. The casting solvents might differently affect the polymer microstructure and polymer-ceramic interface formation both responsible for permeability and IC of the composites. Thus, relying on a strong basis of physicochemical and electrochemical methods, in this section we will discuss the properties of LATP+PVDF membranes as a whole, and specifically of the LATP within. The impact of ball-milling filler preparation on the main structural features of LATP is concisely described in Figure S7, Supplementary Materials.

**Figure 5:**
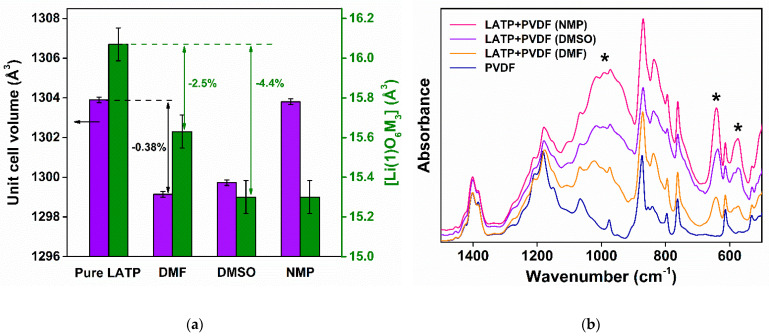
(**a**) Calculated unit cell and [Li(1)O_6_M_2_] polyhedra volumes of the ceramic (pure LATP) and that within the LATP+PVDF composite membranes cast with DMF, DMSO, and NMP; (**b**) ATR−FTIR spectra of PVDF powder and LATP+PVDF membranes cast with the studied solvents. Signals of LATP are marked with asterisks.

Analyzing the structural changes of LATP filler during the membrane fabrication, we can instantly see that they possess a higher order of magnitude than in the still-soaking experiment. Figure 5 shows the LATP cell and its intrastructural polyhedra volumes calculated from the composite membrane XRD analysis, as well as the FTIR spectra of LATP+PVDF. It is seen (Figure 5a) that the fabrication process led to the shrinkage of unit cell volume (*V*) from 1304 to 1299–1300 Å^3^ for DMF- and DMSO-casted membranes in contrast to that of NMP remaining nearly the same. Excluding the NMP, the DMF and DMSO-related shrinkage is two times larger (up to 0.38% losses for DMF) than the largest case for the still-soaking experiment (0.16% losses for DMF, Figure S6, Supplementary Materials). Hence, for the DMF and DMSO cases, we can expect to have strong stoichiometric shifts. Regarding the IC-sensitive [Li(1)O_6_M_2_] polyhedron, it shrinks from 16.0 to 15.6 Å^3^ (2.5% loss) for DMF and to 15.3 Å^3^ for DMSO and NMP (4.4% loss, Figure 5a) indicating drastic changes probably related to a partial Li removing from the intrastructure. For comparison, the biggest change in the soaking part was the 1.0% volume gain for DMF.

It is barely possible to discuss the changes of membrane’s IC in correlation with the LATP structural changes only: the impact of polymer, solvent, and fabrication conditions should be also considered. Indeed, the membrane fabrication process is likely more aggressive to the LATP structure than soaking. A simple example is that an increase of temperature from room to 80 °C can improve the solubility of carbonyls in DMSO [46]. As the membrane slurry mixing is carried out at 60 °C (two fabrication-related factors: enhanced surface area of the ball-milled LATP powder and heat), a much stronger influence of DAs can be reasonably expected. Still, the XRD analysis of composite membranes showed the only, LATP’s, phase, so the FTIR spectroscopy was additionally applied for the phase characterization.

The ATR-FTIR spectra of the LATP+PVDF membranes (Figure 5b) showed the characteristic bands of LATP (broad band due to P–O stretching vibrations centered near 1000 cm^−1^ and O–P–O bending vibrations at ca. 640 and 570 cm^−1^) and those of PVDF (peaks at 614, 762, 838, 870, 1178, and 1400 cm^−1^—see [35] for vibrational assignments). Overall, there was no evident qualitative difference in the spectra — no side signals, which could be ascribed to either residual solvents or undesired products, were detected. Thus, despite some structural fluctuations, none of the three solvents destroyed the filler and polymer phase, and can thus be applied for membrane fabrication.

#### 3.2.2. Morphological Properties, Conductivity, and Permeability of LATP+PVDF

**Figure 6:**
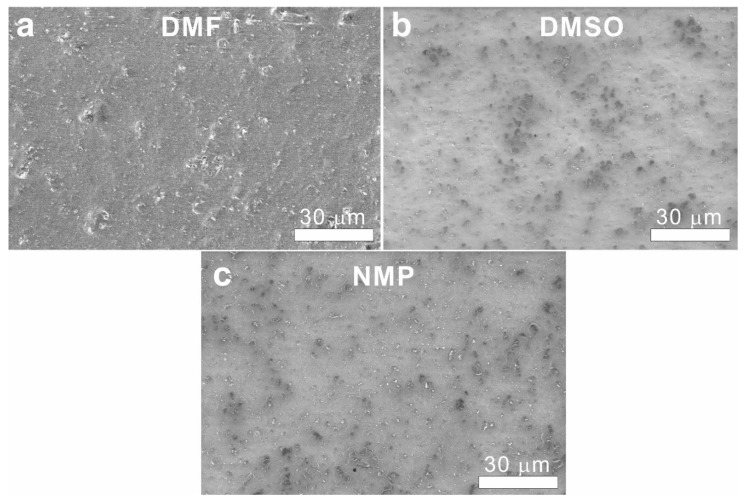
SEM images of the glossy side of a LATP+PVDF membrane fabricated using (**a**) DMF, (**b**) DMSO, and (**c**) NMP solvents.

To study membranes’ morphology, we applied SEM imaging: the glossy sides (which is attached to the substrate during the drying process) of LATP+PVDF are represented in Figure 6, whereas the rough (in contact with air) and cross-section sides are shown in Figure S10, Supplementary Materials. In general, the membrane’s surface is quite smooth, ceramic particles are uniformly distributed, and the LATP’s core element ratios roughly correspond to the theoretical formula units (Figure S11 and Table S4, Supplementary Materials). At the same time, one can see a decent number of pores on the membranes’ surface, especially in the cases of DMSO and NMP solvents (Figure 6b,c), which can be ascribed to a more severe polymer’s globularity (Figure S10, Supplementary Materials). Such a membrane’s morphology impedes its implementation in RFBs, because through pores can be a source of redox species crossover (i.e., membrane’s permeability) [16,34,35,36].

**Figure 7:**
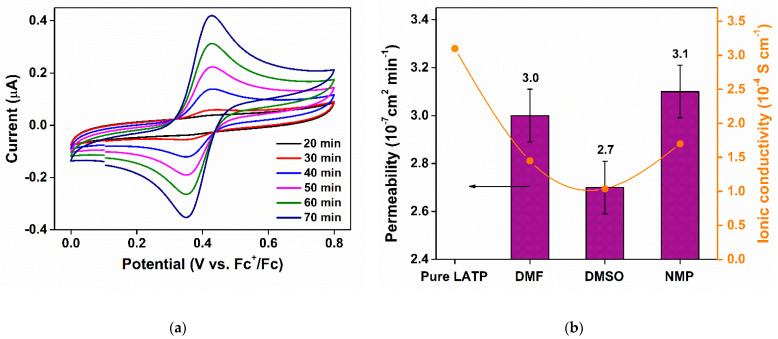
(**a**) Example of time dependent CV trends used for calculation of membrane’s permeability coefficient; (**b**) permeability−IC correlation for LATP+PVDF fabricated using different solvents. Electrolyte composition: 0.5 M TEMPO + 1.0 M LiClO_4_ in PC.

To quantitatively estimate membranes permeability, we used CV and TEMPO, the model redox-active compound frequently exploited in RFB catholytes [47,48,49]. In our set-up (Figure S3, Supplementary Materials), we monitored amount of TEMPO transferring through the membrane from one half-cell to another by recording a CV signal with time (Figure 7a). Previously, we found that permeability of commercially available membranes (i.e., Neosepta, Nafion) in the carbonate-based SE was around 1.0 ∙ 10^−7^ cm^2^ min^−1^. Accordingly, we established the desired permeability for membranes to be the same order of magnitude or lower than 10^−7^ cm^2^ min^−1^ in the current SE. The calculated permeability coefficients were close to each other and equaled 3.0, 2.7, and 3.1 ∙ 10^−7^ cm^2^ min^−1^ for DMF, DMSO, and NMP, respectively (Figure 7b). The important conclusion here is that, in spite of the changes occurred during membrane fabrication, LATP+PVDF permeability did not depend much on the choice of studied solvents. Overall, the calculated permeability coefficients are comparable with the commercially available samples. Still, the values should be further suppressed as much as possible to eliminate the RFB’s capacity decay.

When speaking about RFBs, we should emphasize that membrane’s permeability and apparent IC are linked with each other by means of the membrane’s porosity. In RFBs, the membrane’s porosity system is filled with a liquid electrolyte. It means that apparent membrane’s IC includes the impact of this liquid electrolyte, yet quite small, because the electrolyte’s IC is usually one order of magnitude higher (about 6 ∙ 10^−3^ S cm^−1^) [50,51] than that of ceramics. Nevertheless, the more through pores the membrane has, the higher its total IC. The IC values of LATP+PVDF casted with DMF, DMSO, and NMP were 1.45, 1.04, and 1.70 ∙ 10^−4^ S cm^−1^, respectively (Figure 7b; see Nyquist plots in Figure S12 and Table S5, Supplementary Materials). These values correlate with the permeability data: the DMSO-casted composites possess both the lowest permeability and IC among the studied membranes, whereas the NMP-casted ones have the highest values (Figure 7b). It confirms the connection between IC and permeability. It is also worth mentioning that the IC of obtained membranes are close to that for the ceramics soaked in the solvents (1.6–1.9 ∙ 10^−4^ S cm^−1^, Figure 5b). It likely indicates that the contribution of ceramic to IC of the studied membranes is dominant.

## 4. Conclusions

Pure and well-crystallized LATP samples were successfully synthesized through the solid-state approach. Dense ceramics were shown to possess good IC of 3.1 ∙ 10^−4^ S cm^−1^ that correlate well with previous LATP ceramics studied by our research group. It was first shown that LATP ceramics exhibit instability toward three DA solvents: DMF, DMSO, and NMP. This instability was versatilely represented through a bunch of interrelated changes: morphological (erosion and increased intergranular spacing), slight disturbance of elemental distribution, structural changes, and IC losses. The latter approaches a half of initial IC (1.6–1.9 ∙ 10^−4^ S cm^−1^) after static soaking in the studied solvents. The observed degradation can be primarily explained in terms of the partial and non-stochiometric dissolution of LATP in dipole aprotic solvents (i.e., elution of specific ions as observed in the water-soaking experiment).

Composite membranes consisting of LATP filler and PVDF matrix were fabricated via the tape-casting technique using three studied solvents (DMF, DMSO, and NMP). The filler particles, uniformly distributed within the PVDF matrix, undergo severe structural changes during the membrane preparation, stronger than after the static soaking experiment. The fine powder form and applied heating during the solution mixing are considered as the main factors enhancing the LATP-solvent interaction. Despite this, the LATP’s NASICON phase remained to be the only inorganic unit detectable through the XRD and FTIR techniques. The latter additionally showed the presence of PVDF phase as the only polymer component of the membranes. All membranes were characterized as having a moderate level of porosity that led to the permeability coefficients of 3.0, 2.7, and 3.1 ∙ 10^−7^ cm^2^ min^−1^ for DMF, DMSO, and NMP, respectively. These coefficients correlated with membranes’ IC values of 1.45, 1.04, and 1.70 ∙ 10^−4^ S cm^−1^, respectively, which are quite close to those of the pure LATP ceramic after exposure to the corresponding solvents.

Overall, even though LATP was shown to possess instability toward the DA solvents applied, we lean to the conclusion that DMF, DMSO, and NMP can be still used for the fabrication of LATP+PVDF class of composites. All the solvents led to similar values of the membrane’s permeability—none resulted in a considerable improvement compared to the others. So, new research pathways should be chosen for better suppression of the active species crossover during the operation of RFBs. We hope that the revealed fundamental and applied knowledge regarding ceramic’s stability and composite membrane’s properties will help the involved scientists to continue moving forward the research on RFBs and beyond.

## Data Availability

Not applicable.

## Supplementary Materials

The following supporting information can be downloaded at: www.mdpi.com/article/10.3390/membranes13020155/s1, Figure S1: two-step solid-state synthesis of NASICON-phased Li1.3Al0.3Ti1.7(PO4)3 (LATP) ceramic; Figure S2: fabrication routine (tape-casting method) for LATP+PVDF composite membranes; Figure S3: scheme of two-compartment diffusion cell used for membrane’s permeability evaluation; CE—counter electrode, RE—reference electrode, WE—working electrode; Figure S4: wide-plan SEM images of (a) pristine LATP and that after soaking in (b) DMF, (c) DMSO, and (d) NMP; Figure S5: EDX mapping of LATP ceramic: pristine and after soaking in DMF, DMSO, and NMP; Table S1: EDX analysis of LATP ceramic: mole fractions of Al and Ti in pristine samples and that soaked in DMF, DMSO, and NMP; Table S2: selected characteristic values of LATP crystal structure: *a*, *c*—unit cell dimensions, Å; *V*—unit cell volume, Å^3^; [Li(1)O6M2] and [MO6]—respective intrastructural polyhedra volumes, Å^3^. In brackets – an absolute error corresponded to the last significant digit; Figure S6: the impact of aprotic solvents DMF, DMSO, and NMP on the selected structural features of dense LATP: *a*, *c*—unit cell dimensions; *V*—unit cell volume; [Li(1)O6M2] and [MO6]—respective intrastructural polyhedra volumes; Figure S7: the impact of ball-milling procedure on the selected structural features of as-synthesized LATP: *a*, *c*—unit cell dimensions; *V*—unit cell volume; [Li(1)O6M2] and [MO6]—respective intrastructural polyhedra volumes; Figure S8: schematic representation of the [Li(1)O6M2] and [MO6] intrastructural polyhedra; Figure S9: Nyquist plots of LATP+PVDF membranes casted with DMSO and NMP solvent; dots relate to experimental data and lines—to the fitting curves; Table S3: Corresponding parameters of the equivalent circuit for each fit of the SS/LATP/SS cells. LATP: pristine or soaked in the solvent; Figure S10: SEM images of the cross-section (a–c) and rough (d–f) sides of LATP+PVDF membrane casted with (a,d) DMF, (b,e) DMSO, and (c,f) NMP solvents; Figure S11: EDX mapping of LATP+PVDF composite membranes casted with DMF, DMSO, and NMP solvents; Table S4: EDX elemental distribution of LATP+PVDF composite membranes fabricated using the DMF, DMSO, and NMP solvents. Figure S12: Nyquist plots of the LATP+PVDF membranes casted with DMF, DMSO, and NMP solvent; dots relate to experimental data and lines—to the fitting curves; Table S5: Corresponding parameters of the equivalent circuit for each fit of SS/LATP+PVDF/SS cells. LATP+PVDF fabricated via different solvents (in brackets). Reference [31] is cited in the supplementary materials.
