# Supplementary material for: A Complex Investigation of LATP Ceramic Stability and LATP+PVDF Composite Membrane Performance: The Effect of Solvent in Tape-Casting Fabrication
Source: Membranes (Basel). 2023 Jan 26;13(2):155. doi: 10.3390/membranes13020155 (PMC9965718; doi:10.3390/membranes13020155)
Supplement: Supplementary file 1 [file membranes-13-00155-s001.zip › membranes-2100758-supplementary.pdf]

Supplementary Materials

A Complex Investigation of LATP Ceramic Stability and LATP+PVDF Composite Membrane Performance: The Effect of Solvent in Tape-Casting Fabrication

Zainab Waris [1], Nikita O. Akhmetov [1,*], Mariam A. Pogosova [1], Svetlana A. Lipovskikh [1], Sergey V. Ryazantsev [1] and Keith J. Stevenson [2]

[1] Center for Energy Science and Technology, Skolkovo Institute of Science and Technology, 121205 Moscow, Russia

[2] Department of Chemistry, Lomonosov Moscow State University, 119991 Moscow, Russia

* Correspondence: nikita.akhmetov@skoltech.ru

Table of Contents

Section S1. LATP+PVDF composite membrane fabrication (**Figures S1 and S2**) 2

Section S2. Evaluation of membrane's permeability and ionic conductivity (**Figure S3**) 4

Section S3. SEM & EDX analysis of LATP ceramic (**Figures S4 and S5; Table S1**).................. 6

Section S4. XRD data & crystal structure analysis of LATP (**Figures S6–S8; Table S2**) 9

Section S5. EIS analysis of LATP (**Figure S9; Table S3**) 12

Section S6. LATP+PVDF membranes analysis (**Figures S10–S12, Tables S4 and S5**)............. 13

Section S1. LATP+PVDF Composite Membrane Fabrication (**Figures S1 and S2**)

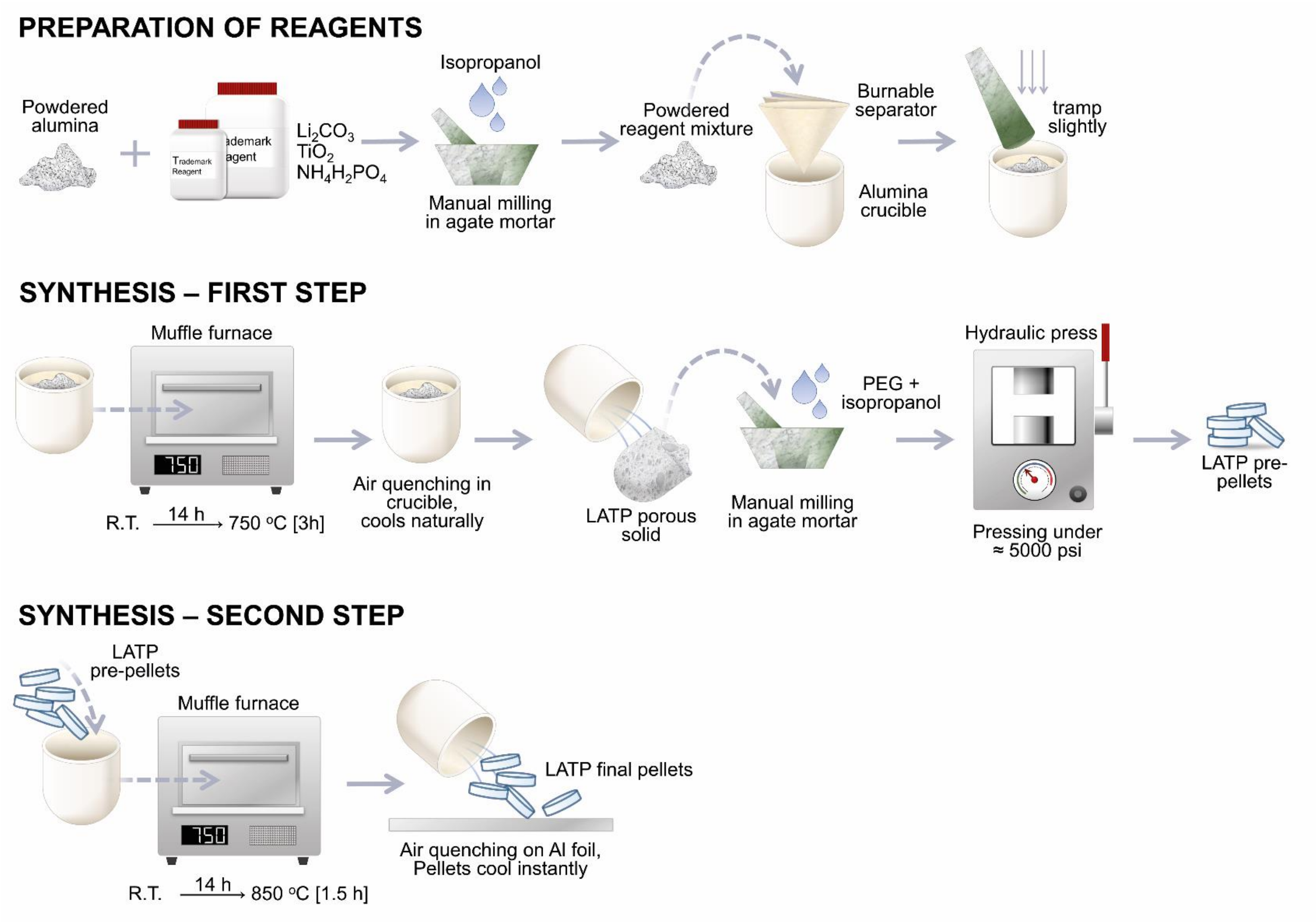

Figure S1. Two-step solid-state synthesis of NASICON-phased $Li_{1.3}Al_{0.3}Ti_{1.7}(PO_4)_3$ (LATP) ceramic.

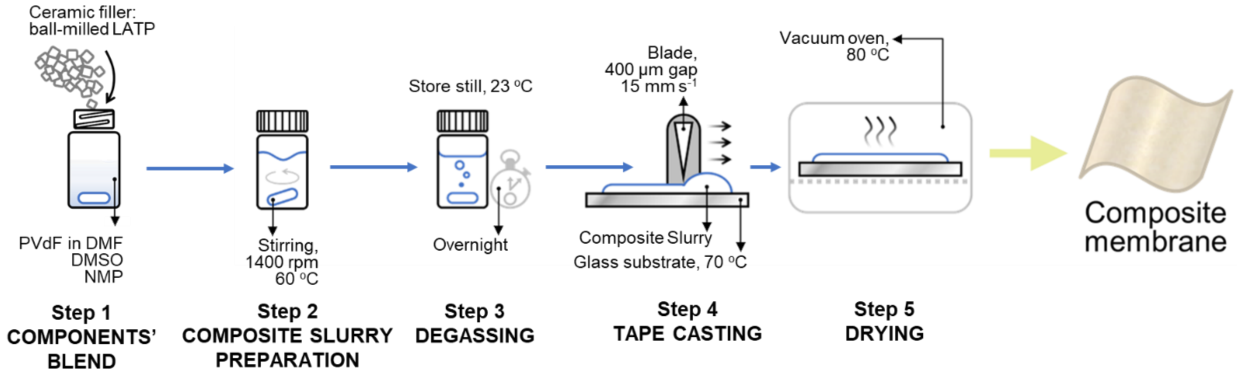

Figure S2. Fabrication routine (tape-casting method) for LATP+PVDF composite membranes.

Section S2. Evaluation of Membrane's Permeability and Ionic Conductivity (**Figure S3**)

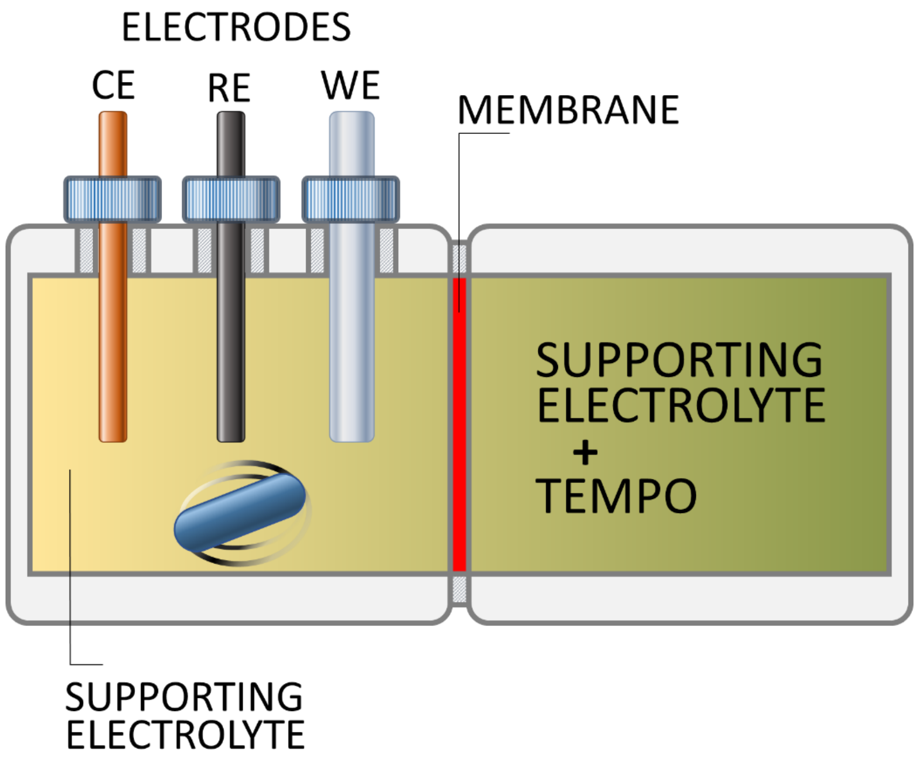

Figure S3. Scheme of two-compartment diffusion cell used for membrane's permeability evaluation; CE – counter electrode, RE – reference electrode, WE – working electrode.

The permeability coefficient (P) was calculated based on obtained cyclic voltammetry (CV) trends by applying the second Fick law to the cylindric geometry of the cell (Equation (S1)):

$$V\frac{dC_l(t)}{dt} = \frac{AP}{L}(C_r(t) - C_l(t)) \quad (S1)$$

where V represents the volume of supporting electrolyte in the left half-cell, $C_l(t)$ is TEMPO concentration in the left half-cell, $C_r(t)$ is TEMPO concentration in the right half-cell, A and L are membrane's area and thickness, respectively.

Using the Randles-Sevcik equation, peak currents (i_p) recorded by CV were recalculated to concentrations (Equation (S2)).

$$i_p = 2.69 \cdot 10^5 n^{3/2} A D^{1/2} V^{1/2} C_l \qquad (S2)$$

where n represents the number of electrons involved in a redox reaction; electrode's area is represented by A; D represents the diffusion coefficient; v represents the scan rate; C_l represents the redox active species concentration.

A series of TEMPO solutions with known concentrations was used to calibrate the peak current response to simplify Equation (S2) to (S3):

$$i_p = K C_l \qquad (S3)$$

where K is the calibration factor, constant in the case of unchanged electrolyte, cell configuration, and scan rate.

Total ionic conductivity (σ_t) of ceramic was calculated based on Equation S4:

$$\sigma_t = \frac{L}{A \cdot (R_1 + R_2)} \qquad (S4)$$

where R_1 is attributed to the bulk resistance, R_2 is attributed to grain boundary resistance, and L and A are thickness and area of a ceramic pellet.

Ionic conductivity of *LATP+PVDF* composite membranes (σ) was calculated via Equation (S5):

$$\sigma = \frac{L}{A R_m} \qquad (S5)$$

where R_m is a membrane resistance and L and A are thickness and area of a membrane, respectively.

Note that as far as we soaked the membrane in a supporting electrolyte (SE) prior to the measurements, the calculated membrane's ionic conductivity is apparent and contain a certain impact of the SE.

Section S3. SEM & EDX Analysis of LATP Ceramic (**Figures S4 and S5; Table S1**).

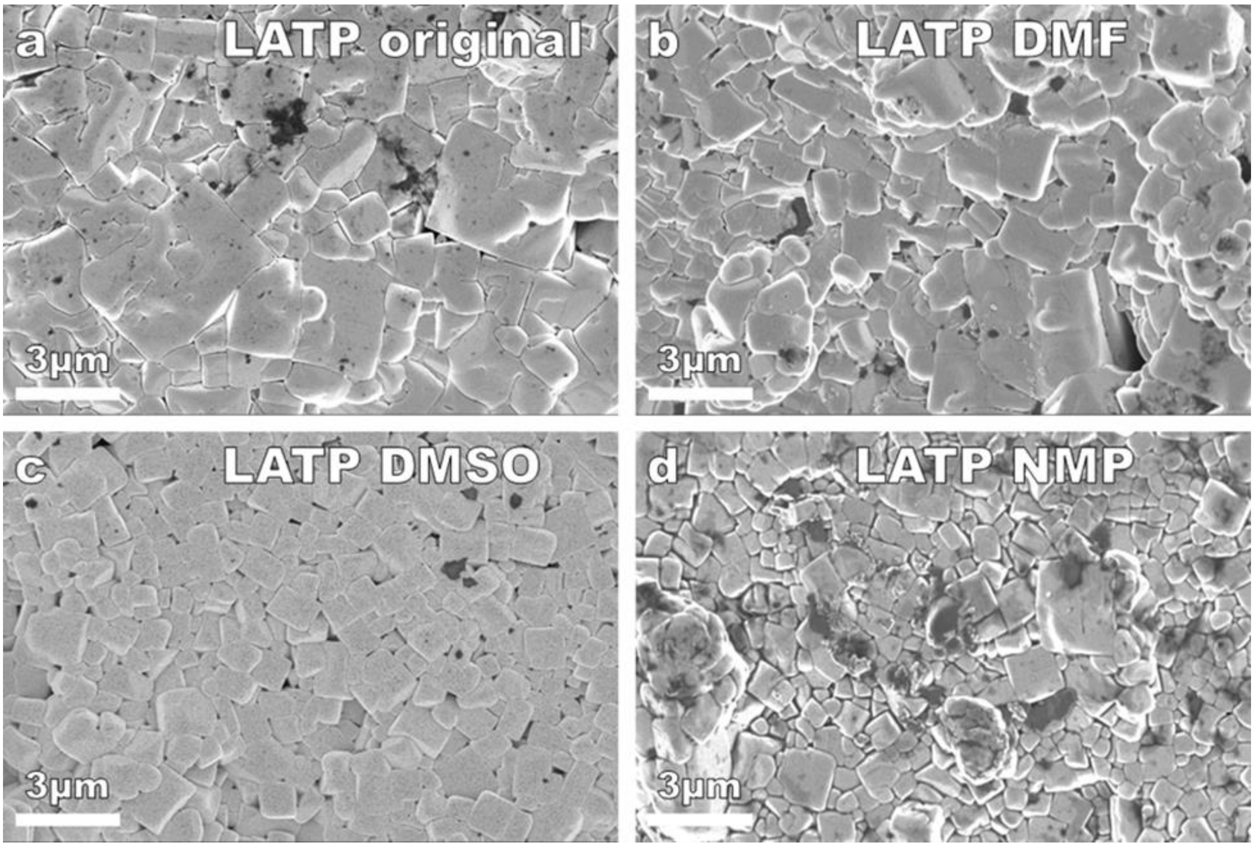

Figure S4. Wide-plan SEM images of (**a**) pristine LATP and that after soaking in (**b**) DMF, (**c**) DMSO, and (**d**) NMP.

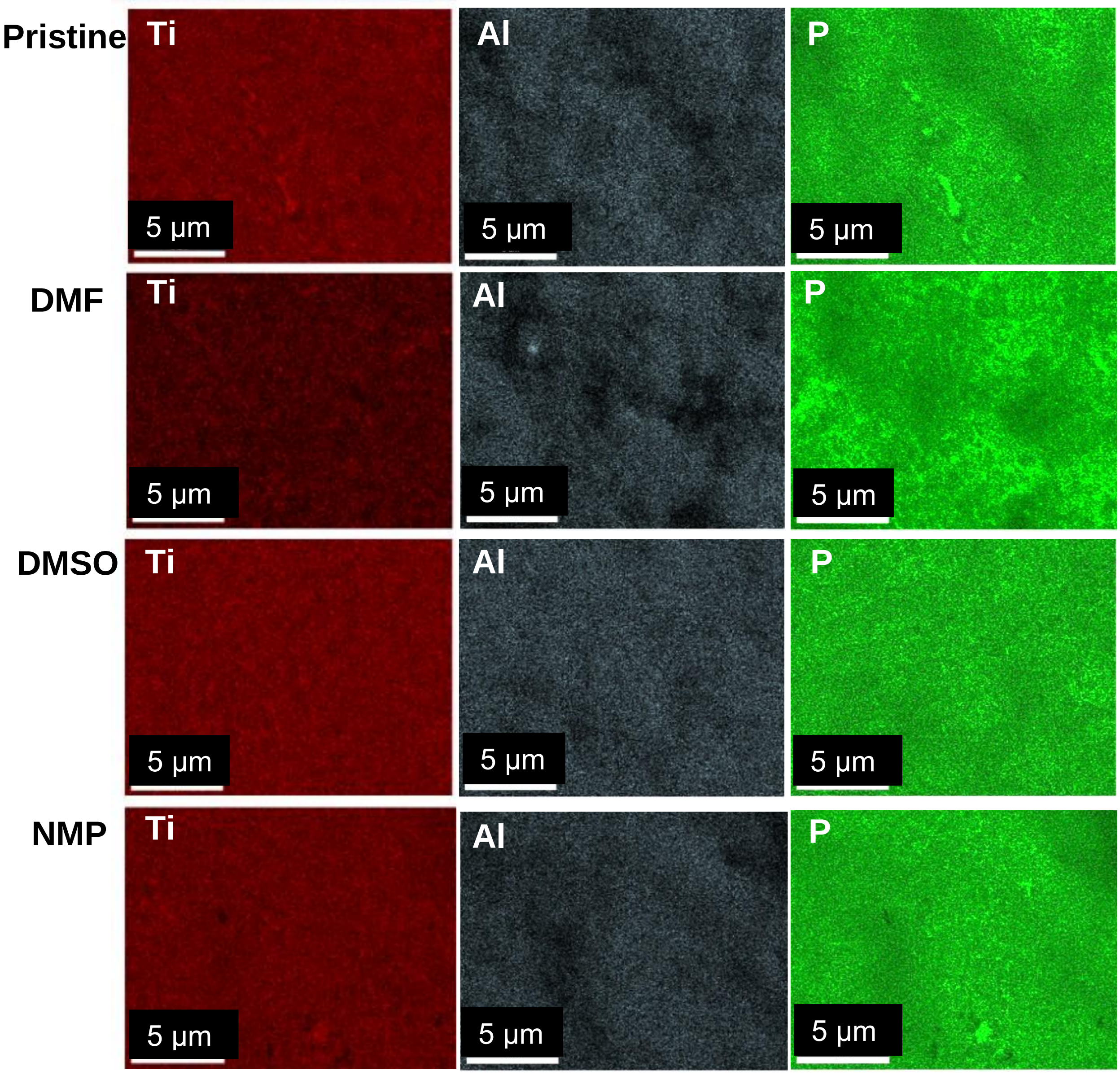

Figure S5. EDX mapping of LATP ceramic: pristine and after soaking in DMF, DMSO, and NMP.

Table S1. EDX analysis of LATP ceramic: the formula unit § of Al and Ti in pristine $Li_{1.3}Al_{0.3}Ti_{1.7}(PO_4)$ samples and that soaked in DMF, DMSO, and NMP.

**Ceramic**	**Location**	**Al**	**Ti**
Pristine ⁎	Average	0.2	1.8
DMF	Average	0.2	1.8
	Edges	0.1	1.9
	Bulk	0.18	1.82
DMSO ⁎	Average	0.22	1.78
NMP ⁎	Average	0.18	1.82

⁎ Values at edges and bulk were close to the average position

§ The formula unit (n_i^{unit}) of the elements of LATP ceramic was determined according to Equation (S6):

$$n_i^{unit} = \frac{5 * C_i^{at.\%}}{\sum_i C_i^{at.\%}} \qquad (S6)$$

where $C_i^{at.\%}$ is atomic percentage of *i* element, whereas "5" represents the sum of theoretical units of Al, Ti, and P elements in the LATP formula (0.3+1.7+3=5).

Section S4. XRD Data & Crystal Structure Analysis of LATP (**Figures S6–S8; Table S2**)

In the current study, LATP ceramic samples from two separate syntheses denoted further as Series 1 and Series 2 were investigated. Accordingly, all changes in structural units were considered and discussed in terms of relative deviations of their values comparing with the reference point obtained in the same synthesis, i.e., within the same Series (Figure S6). For both series, XRD data had been collected for two references: *manually* and *ball-milled* LATP ceramic powders. All treated samples from the first part of this investigation — prolonged soaking of ceramics — were milled manually prior to the XRD data collection, therefore the manually milled reference sample was used. At the same time, as far as we apply a fine LATP powder for membrane fabrication, the ball-milled LATP powder served as a reference in that case. The impacts of the ball-milling procedure on ceramic structure are additionally illustrated in Figure S7. For a comprehensive description of the LATP's sublattice intrastructures, please, refer to [1] and Figure S8.

Table S2. Selected characteristic values of LATP crystal structure: *a*, *c* — unit cell dimensions, Å; *V* — unit cell volume, Å^3; [Li(1)O_6M_2] and [MO_6] — respective intrastructural polyhedra volumes, Å^3. In brackets — an absolute error corresponded to the last significant digit.

	**a**	**c**	**V**	**[Li(1)O_6M_2]**	**[MO_6]**
Series 1					
LATP (manually milled)	8.4977(1)	20.8997(2)	1306.99(3)	15.63(1)	9.36(2)
LATP (ball milled)	8.5004(1)	20.8372(3)	1303.91(2)	15.80(2)	9.31(6)
LATP+DMF (12h)	8.4961(1)	20.8743(3)	1304.92(2)	15.79(2)	9.27(6)
Series 2					
LATP (manually milled)	8.4989(1)	20.8513(3)	1304.34(2)	15.86(2)	9.29(6)
LATP (ball milled)	8.5019(1)	20.8218(3)	1303.42(2)	16.11(2)	9.30(7)
LATP+DMSO (12h)	8.5013(1)	20.8229(2)	1303.30(2)	15.83(2)	9.31(6)
LATP+NMP (12h)	8.5015(1)	20.8229(3)	1303.34(2)	16.00(2)	9.31(6)

[1] Pogosova, M.; et al. *J Power Sources* **2020**, *448*. doi: 10.1016/j.jpowsour.2019.227367

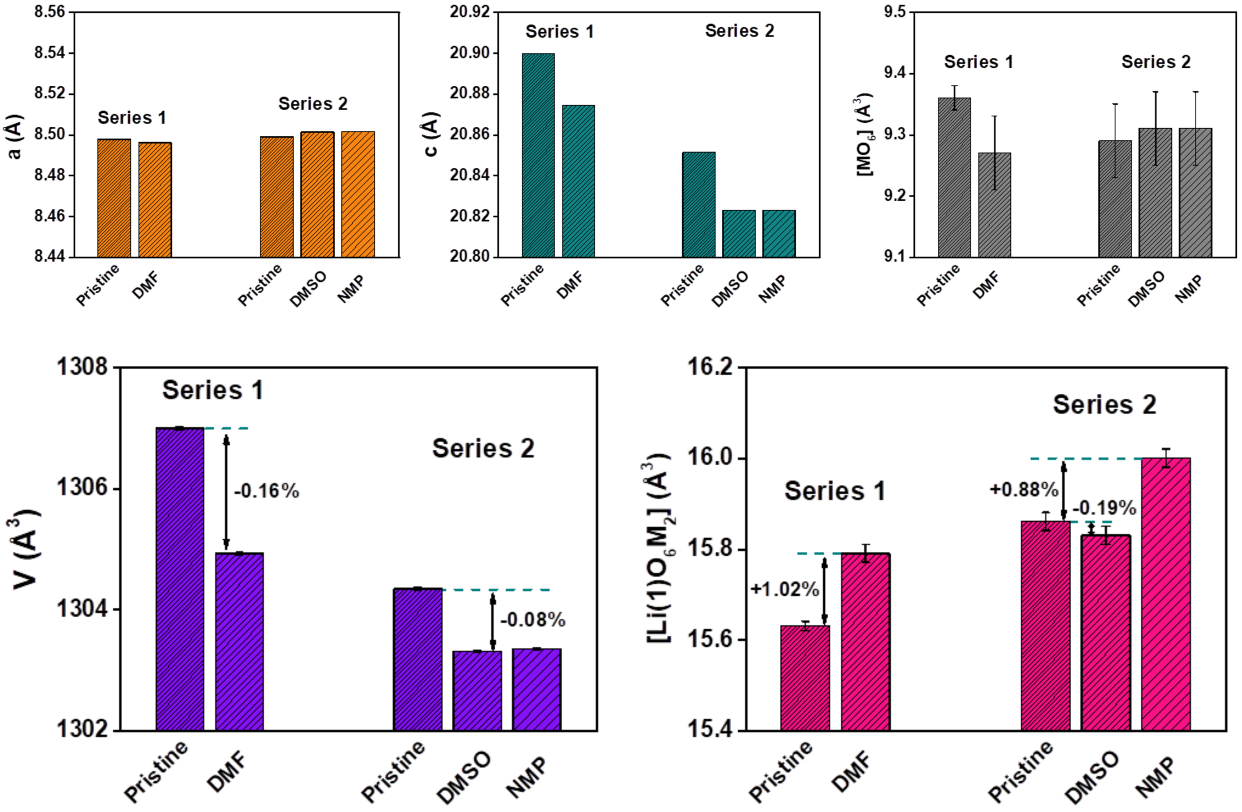

Figure S6. The impact of aprotic solvents DMF, DMSO, and NMP on the selected structural features of dense LATP: *a*, *c* — unit cell dimensions; *V* — unit cell volume; [Li(1)O_6M_2] and [MO_6] — respective intrastructural polyhedra volumes.

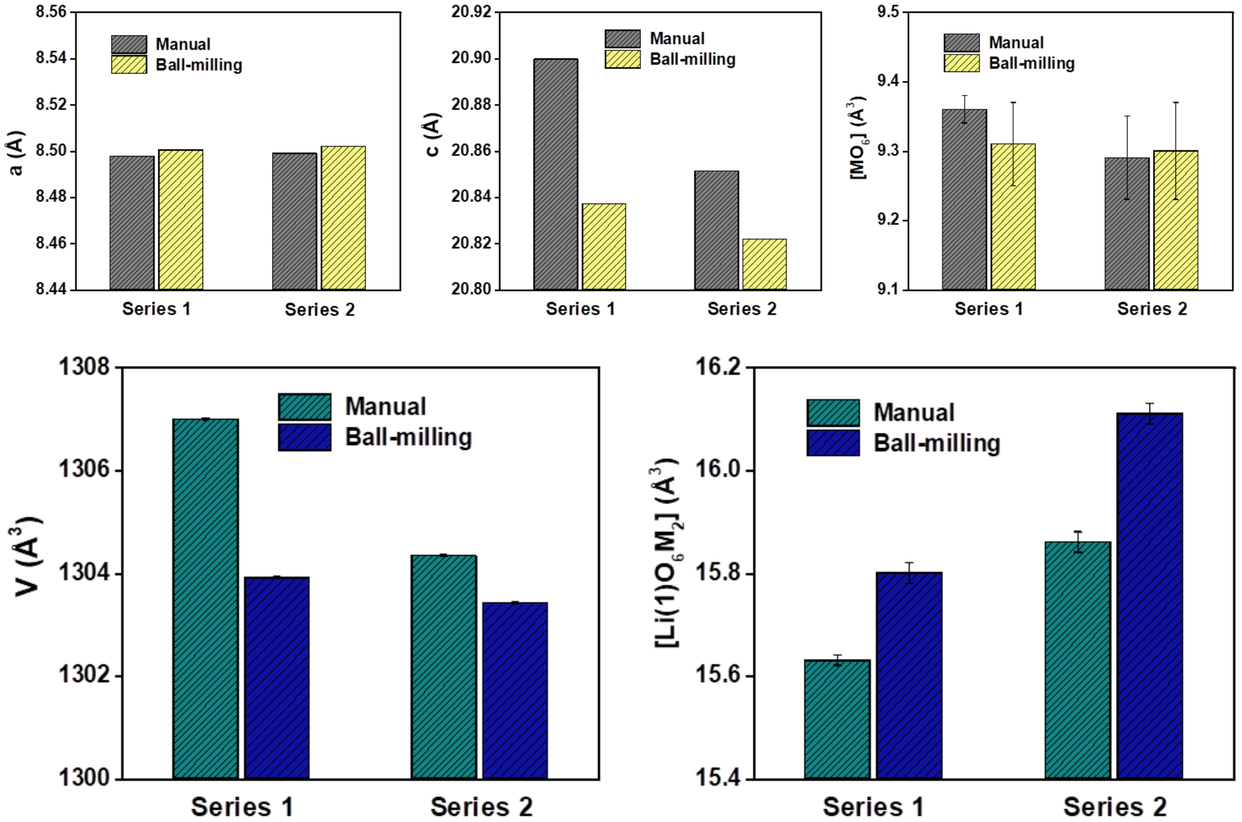

Figure S7. The impact of ball-milling procedure on the selected structural features of as-synthesized LATP: *a*, *c* — unit cell dimensions; *V* — unit cell volume; [Li(1)O_6M_2] and [MO_6] — respective intrastructural polyhedra volumes.

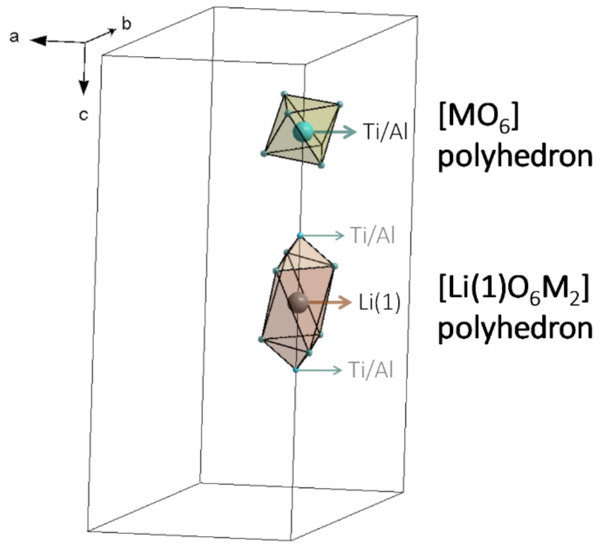

Figure S8. Schematic representation of the [Li(1)O_6M_2] and [MO_6] intrastructural polyhedra.

Section S5. EIS Analysis of LATP (**Figure S9; Table S3**)

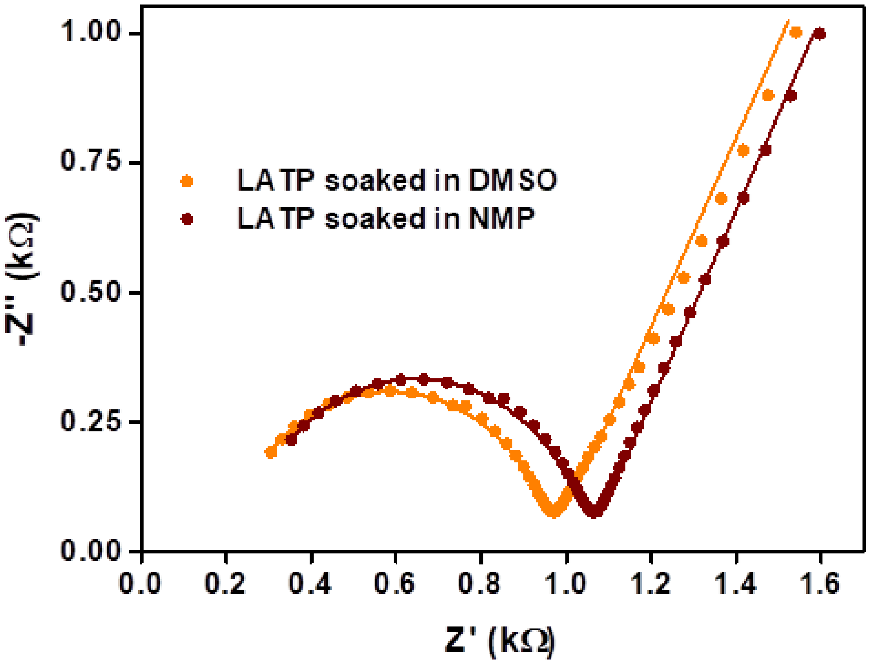

Figure S9. Nyquist plots of LATP+PVDF membranes casted with DMSO and NMP solvent; dots relate to experimental data and lines — to the fitting curves.

Table S3. Corresponding parameters of the equivalent circuit for each fit of the SS/LATP/SS cells. LATP: pristine or soaked in the solvent.

**Ceramic**	**R_b (Ω)**	**R_{gb}**	**Q_{gb} (F s^{n-1})**	**n_{gb}**	**Q_{SS}**	**n_{SS}**
LATP pristine	163	422	$1.09 \cdot 10^{-8}$	0.85	$2.52 \cdot 10^{-5}$	0.79
LATP in DMF	318	910	$3.86 \cdot 10^{-9}$	0.90	$3.65 \cdot 10^{-5}$	0.55
LATP in DMSO	199	764	$8.19 \cdot 10^{-9}$	0.85	$1.59 \cdot 10^{-5}$	0.68
LATP in NMP	242	858	$6.26 \cdot 10^{-9}$	0.86	$3.65 \cdot 10^{-5}$	0.69

Section S6. LATP+PVDF Membranes Analysis (**Figures S10–S12, Tables S4 and S5**).

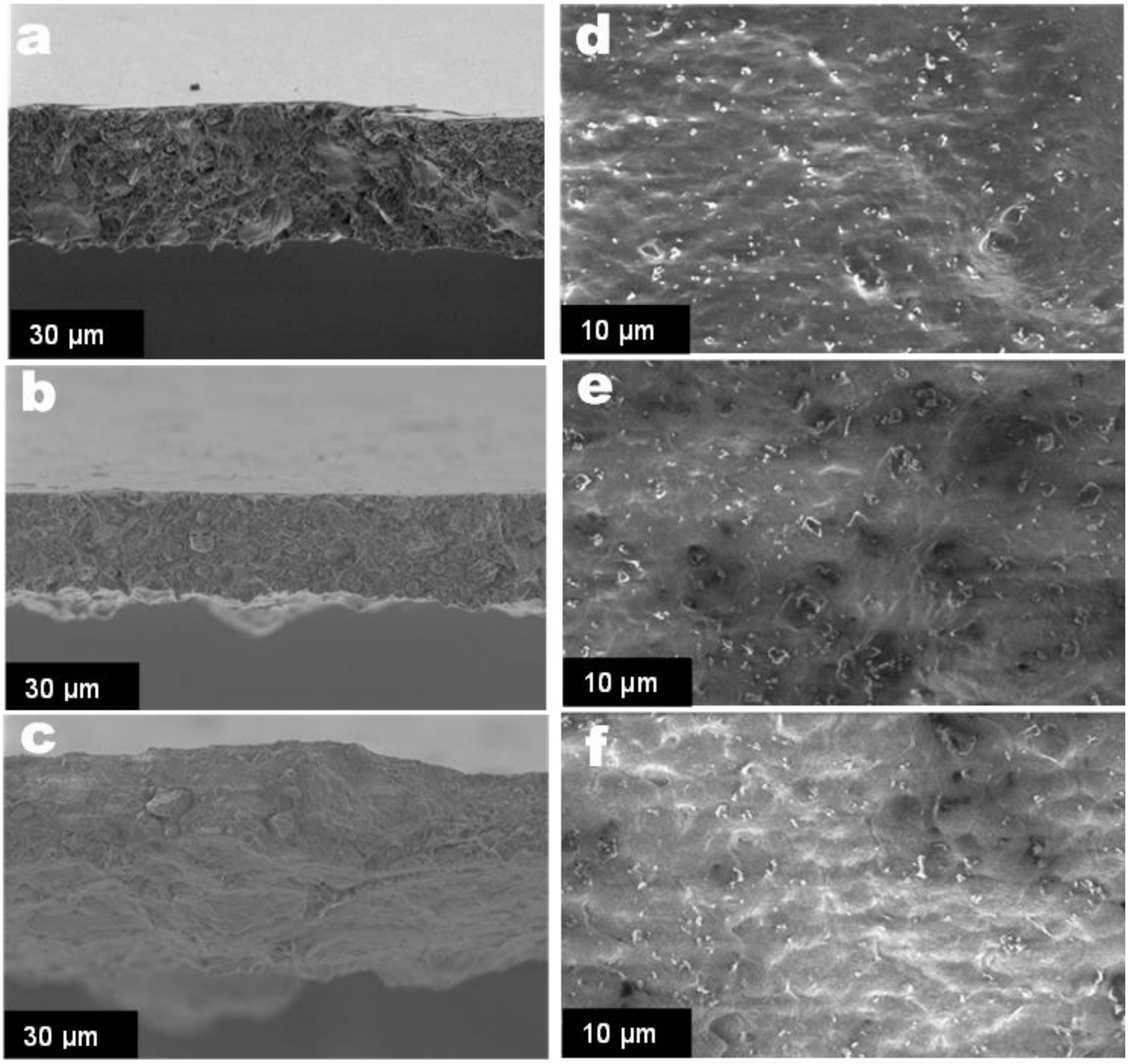

Figure S10. SEM images of the cross-section (**a**–**c**) and rough (**d**–**f**) sides of LATP+PVDF membrane casted with (**a**, **d**) DMF, (**b**, **e**) DMSO, and (**c**, **f**) NMP solvents.

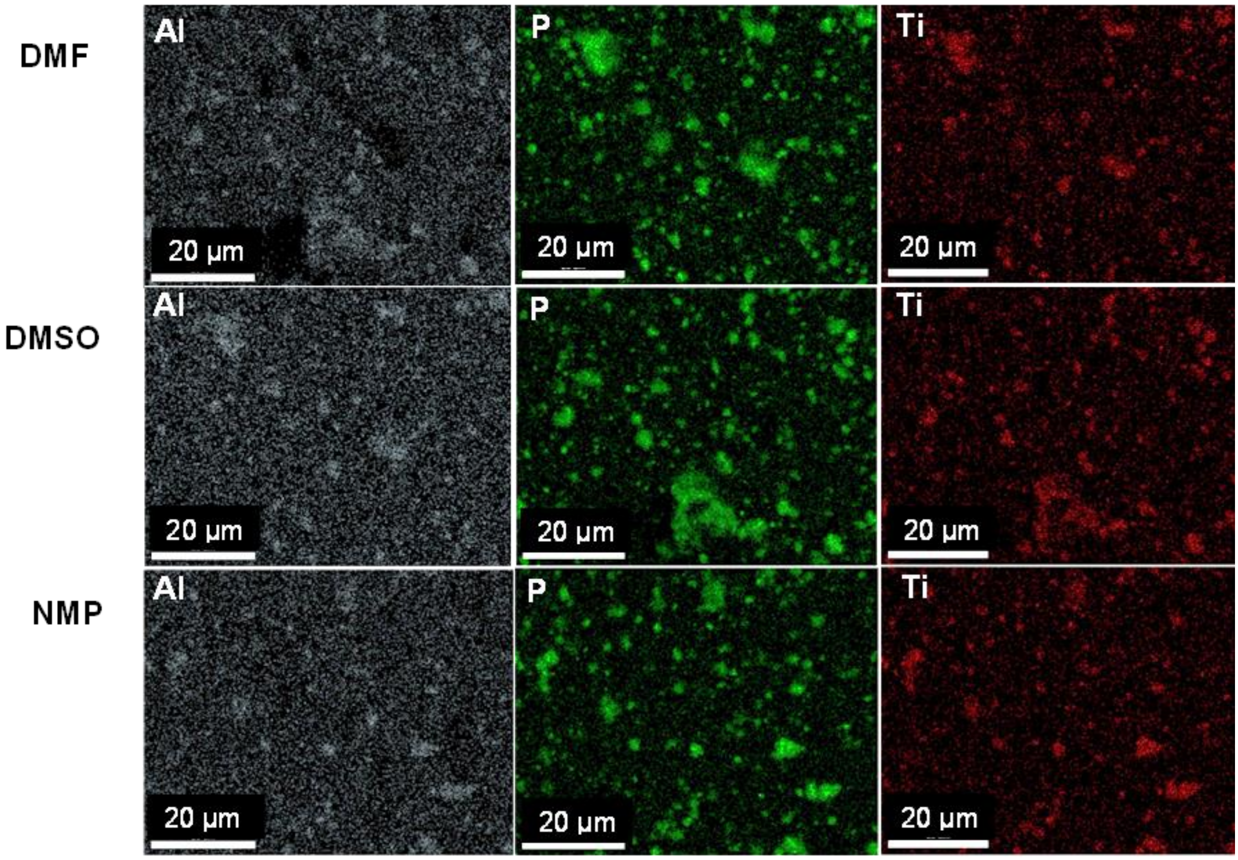

Figure S11. EDX mapping of LATP+PVDF composite membranes casted with DMF, DMSO, and NMP solvents.

Table S4. EDX elemental distribution of LATP+PVDF composite membranes fabricated using the DMF, DMSO, and NMP solvents.

**Solvent**	**Atomic fraction**			**Formula unit**		
	**Al**	**Ti**	**P**	**Al**	**Ti**	**P**
DMF	2.1±0.3	4.4±0.8	11.8±0.9	0.6±0.1	1.2±0.2	3.2±0.2
DMSO	1.9±0.3	4.9±0.8	12.1±0.8	0.6±0.1	1.3±0.2	3.2±0.2
NMP	1.8±0.3	4.7±0.8	11.1±0.9	0.5±0.1	1.3±0.2	3.2±0.2

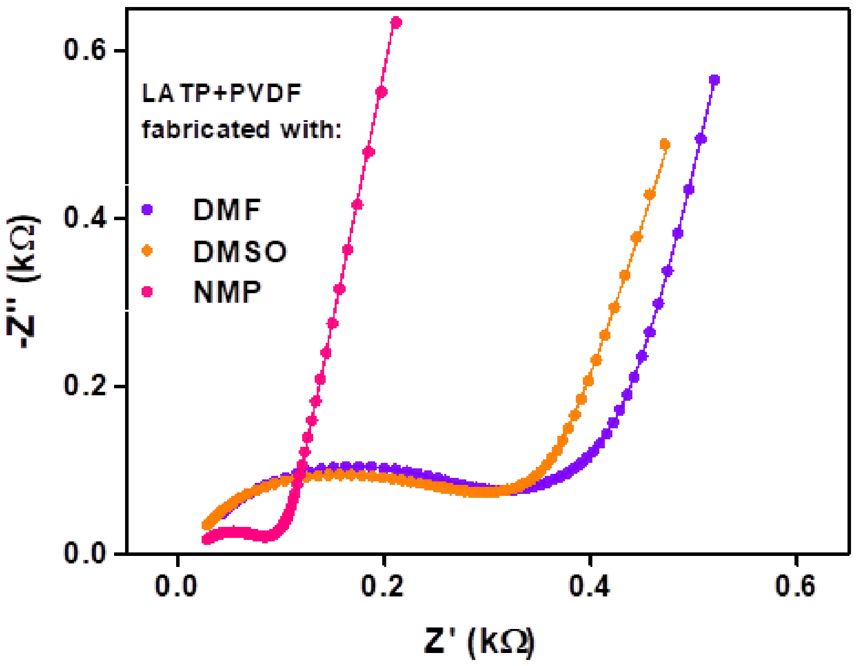

Figure S12. Nyquist plots of the LATP+PVDF membranes casted with DMF, DMSO, and NMP solvent; dots relate to experimental data and lines — to the fitting curves.

Table S5. Corresponding parameters of the equivalent circuit for each fit of SS/LATP+PVDF/SS cells. LATP+PVDF fabricated via different solvents (in brackets).

**Membrane**	**R_m (Ω)**	**R_i**	**Q_i (F s^{n-1})**	**n_i**	**Q_{SS}**	**n_{SS}**
LATP+PVDF (DMF)	12.7	360	$5.33 \cdot 10^{-7}$	0.65	$1.23 \cdot 10^{-5}$	0.77
LATP+PVDF (DMSO)	10.9	322	$5.52 \cdot 10^{-7}$	0.67	$1.39 \cdot 10^{-5}$	0.77
LATP+PVDF (NMP)	15.5	221	$2.5 \cdot 10^{-7}$	0.72	$4.30 \cdot 10^{-6}$	0.91
